# Formulation of Chewable Tablets Containing Carbamazepine-β-cyclodextrin Inclusion Complex and F-Melt Disintegration Excipient. The Mathematical Modeling of the Release Kinetics of Carbamazepine

**DOI:** 10.3390/pharmaceutics13060915

**Published:** 2021-06-21

**Authors:** Adina Magdalena Musuc, Valentina Anuta, Irina Atkinson, Iulian Sarbu, Vlad Tudor Popa, Cornel Munteanu, Constantin Mircioiu, Emma Adriana Ozon, George Mihai Nitulescu, Mirela Adriana Mitu

**Affiliations:** 1 “Ilie Murgulescu” Institute of Physical Chemistry, Romanian Academy, 202 Spl. Independentei, 060021 Bucharest, Romania; iatkinson@icf.ro (I.A.); vtpopa@icf.ro (V.T.P.); munteanuc@icf.ro (C.M.); 2 Department of Physical and Colloidal Chemistry, Faculty of Pharmacy, “Carol Davila” University of Medicine and Pharmacy, 020956 Bucharest, Romania; valentina.anuta@umfcd.ro; 3 Department of Pharmaceutical Physics and Biophysics, Drug Industry and Pharmaceutical Biotechnologies, Faculty of Pharmacy, “TituMaiorescu” University, 004051 Bucharest, Romania; 4 Department of Applied Mathematics and Biostatistics, Faculty of Pharmacy, “Carol Davila” University of Medicine and Pharmacy, 020956 Bucharest, Romania; constantin.mircioiu@umfcd.ro; 5 Department of Pharmaceutical Technology and Biopharmacy, Faculty of Pharmacy, “Carol Davila” University of Medicine and Pharmacy, 020956 Bucharest, Romania; mirela.mitu@umfcd.ro; 6 Department of Pharmaceutical Chemistry, Faculty of Pharmacy, “Carol Davila” University of Medicine and Pharmacy, 020956 Bucharest, Romania; george.nitulescu@umfcd.ro

**Keywords:** carbamazepine, β-cyclodextrin, release kinetics, inclusion complex, F-Melt excipient, chewable tablets

## Abstract

Due to its low solubility, carbamazepine (CBZ) exhibits slow and incomplete release in the gastrointestinal tract and, hence, variable pharmacokinetics and pharmacodynamic effect. Lots of methods have been devised to improve its solubility, the large number of proposed solutions being a sign that the problem is not yet satisfactorily solved. The persistent problem is that predictable release kinetics, an increased rate but within defined limits, are required to avoid high absorption variability. This paper presents a synthesis of a carbamazepine-β-cyclodextrin inclusion complex (CBZ-β-CD), the characterization of the physical mixture, CBZ, β-CD and the CBZ-β-CD inclusion complex using Fourier transform infrared spectroscopy, scanning electron microscopy, simultaneous thermal analysis and X-ray diffraction, formulation of chewable tablets, determination of the dissolution of carbamazepine in medium containing 1% sodium lauryl sulfate (LSS), and in simulated saliva (SS), mathematical modeling of release kinetics. The kinetics of total CBZ release from tablets containing CBZ-β-CD and super-disintegrant F-Melt in both SS and LSS followed two steps: a burst release in the first minutes and a slower release in intervals up to 60 min. The release in the second phase has been well described by the Higuchi and Peppas models, which advocate a controlled release by combined diffusion and with some phenomena of swelling and relaxation of the matrix generated by the crospovidone component of the F-Melt excipient.

## 1. Introduction

Carbamazepine (CBZ) is a medication used primarily in the treatment of epilepsy. Carbamazepine was discovered in 1953 and it was authorized in therapy in 1962. Although many new drugs addressing to epilepsy appeared in the next 50 years, carbamazepine remained one of the most effective drugs, being included on the World Health Organization’s List of Essential Medicines [1]. The problem of increasing solubility of active substances is old as pharmacy itself, and the efforts focused on carbamazepine were a continuous concern of pharmacists in the last fifty years. In fact, the increasing of solubility is essentially a method for improving in vitro availability and also implications for in vivo availability [2,3,4]. In this direction, a long series of research contributions tried to increase the solubility of CBZ, particularly by its inclusion in cyclodextrins.

The extent of absorption of carbamazepine from a carbamazepine-2-hydroxypropyl-β-cyclodextrin complex was significantly greater, and the rate of absorption was faster when compared with an immediate-release carbamazepine tablet in dogs [5].

The improvement of the aqueous solubility of CBZ using cyclodextrins (CDs) and developing an aqueous parenteral formulation was successfully accomplished [6]. Injectable carbamazepine solution obtained by complexing with 2-hydroxypropyl-β-cyclodextrin (HP-β-CD) was tested in terms of pharmacokinetics in vivo in dogs.

An improvement of oral bioavailability of carbamazepine suspension by inclusion in 2-hydroxypropyl-β-cyclodextrin was obtained [7]. The extent of absorption in rats was higher than that of pure drug. Oral solution formulations based on CBZ-CD were also compared with commercially available tablets and suspensions concerning the pharmacokinetics in dogs [8].

Physical–chemical characteristics of carbamazepine-cyclodextrin inclusion compounds and carbamazepine–polyethylene glycol solid dispersions were studied concerning the dissolution profiles in 0.1 N HCl medium. Inclusion complexes with cyclodextrins demonstrated a faster dissolution rate in comparison with solid dispersions and CBZ alone [9]. Dissolution of CBZ included in different carriers such as polyethylene glycols (PEG), phospholipids and hydroxypropyl-β-cyclodextrin (HP-β-CD), and in vivo areas under the CBZ concentration–time curves were significantly higher than those resulting after Tegretol^®^ suspension [10].

Inclusion complexes of CBZ were also prepared with α-CD, β-CD and di-O-methyl-β-cyclodextrin (DM-β-CD). The bioavailability and anti-convulsion activity of CBZ were determined in animals following oral administration of the prepared complexes and compared with the sole drug [11].

*CBZ-CD Tablets*. A first formulation of CBZ-CD tablets was performed more than twenty years ago. The effect of β-cyclodextrin and 2-hydroxypropyl-β-cyclodextrin on the physical properties and dissolution rate of CBZ tablet samples was studied [12]. Later, the release of a CBZ-CD complex from hydroxypropylmethylcellulose (HPMC) matrix tablets was evaluated [13]. The study demonstrated the improvement of CBZ aqueous solubility by adding increasing amounts of β-CD. The bioavailability of the complex from HPMC matrix tabletswas evaluated in Beagle dogs [14]. The stability constant calculated from the phase solubility diagram indicated that the CBZ-CD complexes were adequately stable [15].

Polyethylene glycol 6000 (PEG-6000) was used for the formulation of immediate release tablets [16], and HPMC for the formulation of extended-release tablets [17]. Formulations of fast dissolving tablets of carbamazepine obtained by direct compression technique of β-cyclodextrin complexes and various super disintegrants, such as Indion-414, croscarmellose sodium, crospovidone and sodium starch glycolates were characterized by differential scanning calorimetry (DSC), Fourier transform infrared spectroscopy (FTIR) and stability studies [18].

The formulations prepared with mannitol solid dispersion presented disintegration time in the range of 11.83–17.79 s. However, the formulations prepared with PEG-6000 and polyvinylpyrrolidone (PVP) solid dispersions did not disintegrate within specified limits of time for the fast-dissolving tablet [19]. Whatever the time variation of dissolution, an initial, more or less short burst release was present in all cases.

A series of experiments were performed for the development of fast dissolving tablets. Croscarmellose was used as disintegrant and different excipients for preparing solid dispersions. Complex generic drugs are generally not bio-equivalent with the reference products: therefore, the increase in number of marketed drug/cyclodextrin formulations is particularly slow [20].

The present research involved, in the first step, the synthesis of a carbamazepine-β-cyclodextrin inclusion complex (CBZ-β-CD); the formation of the inclusion complex was confirmed using Fourier transform infrared spectroscopy (FTIR), scanning electron microscopy (SEM), simultaneous thermal analysis (STA) and X-ray diffraction (XRD); finally, the formulation of chewable tablets included the complex and F-Melt excipient. The second step involved a study of the CBZ dissolution in medium containing 1% sodium lauryl sulfate (LSS) and in simulated saliva (SS), the mathematical modeling of release kinetics, and the estimation of the release mechanism. Even though there are many published reports, the problems of inclusion of carbamazepine in several cyclodextrines’ cavities have not been solved [21,22,23,24]. The novelty of the present study consists in the obtained chewable tablets as a final product. The chewable tablets are a special type of tablet, as described by European Pharmacopoeia, which have different characteristics and dissolution performances compared with the conventional ones. Such tablets were manufactured within the present study, and their dissolution profile was investigated. The choice of β-CD as host molecule instead of other cyclodextrins was based on its typical features: (i) as the therapeutical dose of carbamazepine is 200 mg per tablet, a 1:1 inclusion complex with HP-β-CD would need a quantity of 1304 mg. That would involve high-weight tablets (minimum 2 g) with a content of 1.5 g of active ingredient. Such tablets are hardly accepted by patients and have not proven to be stable; (ii) β-CD has a better flowability than HP-β-CD and, being the main constituent of the final product, it was important to choose the best cyclodextrin in terms of flowing and compressibility. As the most abundant ingredient used in the tablets’ formulation is the inclusion complex, the kneading method, with no addition of any solvent, was chosen for obtaining this complex. Using “non-solid state” methods would involve supplementary moisture in the powder, with important decreasesin its flowability and compressibility, and eventually a drastic alteration of the physical–chemical parameters of the chewable tablets.

## 2. Materials and Methods

### 2.1. Materials

Carbamazepine was purchased from Baoji Guokang Bio-Technology Co., Ltd., (Baoji, China). β-cyclodextrin was obtained from Global Holding Group Co., Ltd., (Ningbo, China). The ethanol and distilled water were of analytical grade. F-Melt^®^ was purchased from Fuji Chemical Industries Co., Ltd. (Toyama, Japan).

For the weighing of the substances, a Mettler Toledo AT261 balance (with 0.01 mg sensitivity) was used.

### 2.2. Physical Mixture and Inclusion Complex Synthesis

The inclusion complex of CBZ with β-CD (in 1:1 molar ratio) was obtained by the kneading method of complexation in solid state. This molar ratio was chosen on technical requirements for obtaining the final product, i.e., acceptable weight, oral dispersion and mechanical properties for both the compressible powder and chewable tablets. The actual formation of the 1:1 inclusion complex was proved by post synthesis (kneading) physical–chemical characterization and by dissolution experiments. The physical mixture maintaining the same 1:1 molar ratio was used as reference. In the present study, it was not necessary to calculate a stability constant because the lower 1:1 molar ratio was chosen due to reasons related to the tablet weight requirements. Available literature data [13,14,15,21] indicated values of *K*_s_ of 376.5–636 M^−1^ for various phase solubility diagrams of A_L_ type. All reported phase solubility diagrams exhibited slopes of less than 1, proving the existence of a 1:1 molar ratio complex.

For the physical mixture, the necessary amounts of the raw components were sieved and accurately weighted (to yield 1:1 molar ratio) and then physically mixed for 15 min in an agate mortar, at the room temperature, in order to obtain a homogeneous powder.

For the inclusion complex synthesis by kneading, 0.25 mmol of CBZ and 0.25 mmol of β-CD, in 1:1 molar ratio, were thoroughly mixed together in a mortar, with vigorous trituration, for about 3 h. During this process, an appropriate volume of 70% ethanol solution was added until a homogeneous paste was obtained. The resulting paste was further triturated for 1 h. Then, the obtained product was dried, at room temperature, for 24 h. The solid dried mixture was passed through sieve no. VI [25]. The solid dispersion was stored in the desiccator, over anhydrous calcium chloride, until use.

The formulation of chewable tablets with 200 mg of CBZ, the exact composition, the used pressure and their pharmacotechnical characterization were performed as described in a previous paper [26]. The powder for direct compression contained the active ingredient—the inclusion complex CBZ-β-CD at a molar ratio of 1:1—and F-MELT^®^, Fuji’s patented F-MELT^®^ system, which was specifically designed for Oral Disintegrating Tablets.

### 2.3. Characterization

Pure CBZ and β-CD, the physical mixture and the inclusion complex were evaluated by Fourier transform infrared spectroscopy (FTIR) (Jasco International Co. Ltd., Tokyo, Japan), scanning electron microscopy (SEM) (Thermo Fisher, Waltham, MA, USA), simultaneous thermal analysis (STA) (Netzsch-Geratebau GmbH, Selb, Germany) and X-ray diffraction (XRD)(Rigaku, Tokyo, Japan). FTIR spectra were recorded using a JASCO FT/IR-4200 spectrometer equipped with an ATR PRO450-S accessory (Jasco International Co. Ltd. Tokyo, Japan). The spectra were collected in the spectral range from 4000 to 400 cm^−1^, with the resolution of 4 cm^−1^. The spectra are presented in transmittance percentages (T%) versus wavelength (cm^−1^). Scanning electron microscopy images were carried out in a FEI Quanta 3D FEG microscope (Thermo Fisher, Waltham, MA, USA). SEM images were acquired under magnification of 1000× to 4000×. Powder X-ray diffraction patterns were recorded using an X-ray diffractometer (RigakuUltima IV diffractometer), with CuKα radiation source (λ = 1.5406 Å), in the 2θ = 5–60° range. The used scan speed was 5°/min and a step size of 0.02°, at 40 kV and 30 mA. For phase identification, a Rigaku’s PDXL software connected to ICDD PDF-2 database was used. The thermal behavior of pure compounds, physical mixture and the inclusion complex was studied in a dynamic argon atmosphere flow of 40 mL/min using a Netzsch STA 449 F1 Jupiter simultaneous thermal analyzer in the range of 30–600 °C, at a heating rate of 5 °C/min. The accurately weighed samples (the precision of the balance is 0.1 mg) were placed in Al_2_O_3_ pans without lids. All measurements were performed in duplicate.

While the formation of complexes is possible by many methods, the efficiency of embedding is different from one method to another, as proved by thermogravimetric analysis (TG) and DSC analysis in the case of inclusion of meloxicam in β-CD [27].

### 2.4. Quantitative Analysis of CBZ

The quantitative analysis of carbamazepine was carried out using a Waters liquid chromatographic system (Waters, Milford, MA, USA) consisting of a 600 E Multisolvent Delivery System, Waters AF degasser, 486 UV tunable absorbance detector and Waters 717 plus automated sample processor. Empower Pro software (Waters) was used to control the instrument, data acquisition and processing.

The chromatographic separation was performed using a Hypersil Gold, 5 μm 150 × 4 mm column (Thermo Fisher Scientific, Waltham, MA, USA) maintained under constant temperature (30 °C). The mobile phase consisted of an isocratic mixture of 0.1% trifluoroacetic acid-acetonitrile (60:40 *v*/*v*), delivered at 1.0 mL/min flow rate. The detector was set at 285 nm, and the injection volume was 5 μL. All solutions were filtered through a 0.45 μm pore size filter (LABTECH VP30) and degassed by sonication.

The HPLC method was subjected to validation in accordance with the International Conference on Harmonization (ICH) regulations Q2 (R1) in terms of specificity, linearity, precision (repeatability and intermediate precision) and accuracy [28].

The linearity assessment was performed using six carbamazepine concentration levels, in the range of 3.125–100 μg/mL. All analyses were performed in triplicate.

The detection limit (LOD) and quantitation limit (LOQ) were determined based on the signal-to-noise ratio. The concentrations yielding to signal-to-noise ratios of 3:1 and 10:1 were considered as the LOD and LOQ, respectively.

Precision was evaluated for repeatability and intermediate reproducibility on spiked quality control (QC) samples, at three different concentration levels (QClow—8 μg/mL; QCmedium—20 μg/mL; QChigh—80 μg/mL). The RSD% values computed for absolute peak are as resulting from interpolation on the corresponding calibration curves were considered as precision indicators. Repeatability study was performed by injection of five replicates from a single prepared spiked sample within a single experimental session, whereas intermediate reproducibility was tested by means of five different samples processed in different experimental sessions to ascertain the QC level. The bias (%) between the concentration values determined for the QC samples and their nominal values was used as an accuracy indicator.

### 2.5. In Vitro Release Kinetics Studies

The release kinetics of carbamazepine from the experimental chewable tablets containing the CBZ-β-CD inclusion complex were performed on a DT 800H dissolution tester (Erweka, Langen, Germany). The dissolution test was performed using the USP 32 specifications [29], with the Apparatus 2 (paddles) at 75 rpm and 900 mL of 1.0% sodium lauryl sulfate (LSS) aqueous solution at 37.0 ± 0.5 °C, as dissolution medium.

Since the CBZ-β-CD inclusion complex was formulated into chewable tablet, a second dissolution medium, simulating the physical–chemical properties of saliva, was also tested. The final pH of this medium, which contains 8 g/L NaCl, 0.19 g/L KH_2_PO_4_, 2.38 g/L Na_2_HPO_4_, is 6.8 [30,31]. Both the experiments in 1% LSS and in simulated saliva were run on 12 tablets. Samples (2 mL) were removed after 5, 10, 15, 30, 45 and 60 min in a glass syringe, filtered through a 0.45-µm Teflon^®^ filter, and diluted 1:10 with methanol. Quantification of the released carbamazepine was performed using an HPLC method, with UV detection at 285 nm.

Dissolution profiles were compared using the *f*_2_ similarity factor [32]:

(1) $$f_{2}=50\log\left\{{\left[1+\frac{\sum_{i=1}^{p}{\left(\bar{x_{ti}}-\bar{x_{ri}}\right)}^{2}}{P}\right]}^{-1/2}\times 100\right\}$$

where *ri* and *ti* are the average percentage of carbamazepine dissolved at a specific time from the reference and test products, respectively, and *P* is number of measured time points used to evaluate the amount of dissolved carbamazepine.

In order to evaluate the mechanism of the carbamazepine release kinetics, dissolution data were fitted to different kinetic models: zero order, first-order, Higuchi, Korsmeyer–Peppas and Weibull [33,34,35,36].

## 3. Results and Discussion

### 3.1. Organoleptic Evaluation of the Compounds

The color, smell and taste of the tablets were evaluated according to the European Pharmacopoeia specifications [37]. Both the physical mixture and the inclusion complex, obtained by the kneading method, were white, crystalline and odorless powders, with a bitter taste.

### 3.2. Physical–Chemical Characterization of Compounds

**Figure 1:**
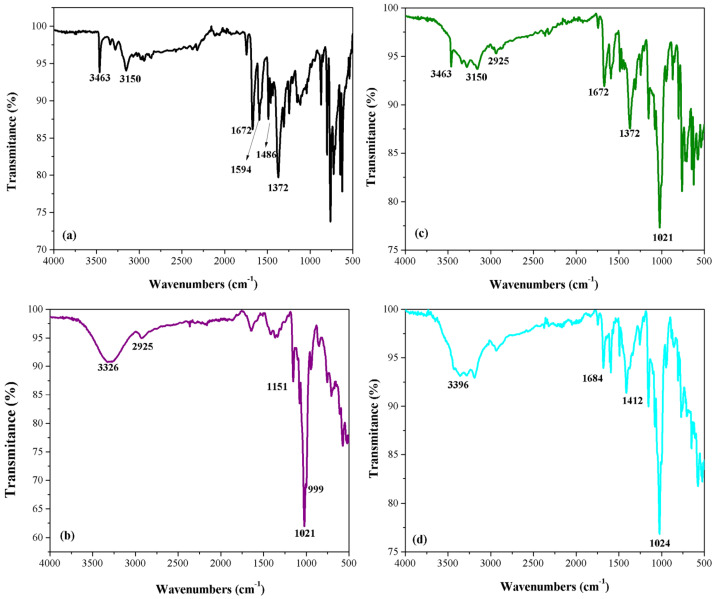
FTIR spectra of (**a**) CBZ, (**b**) β-CD, (**c**) CBZ-β-CD physical mixture and (**d**) CBZ-β-CD inclusion complex.

*FTIR spectroscopy.* Figure 1 shows the FTIR spectra recorded for the (a) carbamazepine, (b) β-cyclodextrin, (c) physical mixture of carbamazepine and β-CD and (d) inclusion complex of carbamazepine-β-cyclodextrin.

The FTIR spectrum of carbamazepine (Figure 1a) presents one narrow band at 3463 cm^−1^, produced by N–H stretching frequencies from the primary amine group bond’s vibration. Aromatic C–H stretching vibrations occur at 3150 cm^−1^. A weak absorption band appears at 1240 cm^−1^ which is assigned to C–N stretching vibration. The peak at 1372 cm^−1^ is assigned to N–H deformation. The C=C ring stretching vibration occurs at 1486 cm^−1^. The C=O bond of the amide group gives a medium intensity band at 1672 cm^−1^ (Figure 1a). A slight difference in wavenumber as compared with literature data is observed [38], but all the data confirm the presence of CBZ in Form III.

The FTIR spectrum of β-CD (Figure 1b) shows a large band between at 3000 and 3600 cm^−1^, due to the strong O–H stretching vibrations from the primary or secondary hydroxyl groups. The peaks at 2925 cm^−1^ and 1151 cm^−1^ are attributed to the symmetric and antisymmetric stretching of the C-H bond in the CH and CH_2_ groups and ν[C–C], respectively. A strong band at 1021 cm^−1^ and a shoulder at 999 cm^−1^ are produced by the O–H bending vibration and C–O bond vibration (Figure 1b) [39,40]. The FTIR spectrum of the physical mixture (Figure 1c) displays all characteristic peaks for β-CD that overlap with the characteristic carbamazepine peaks. Their frequencies and intensities remained practically unaltered. The FTIR spectrum of the carbamazepine-β-CD inclusion complex (Figure 1d) presents clear spectral changes in comparison with the two separate substances and with their physical mixture. The characteristic spectral portion for carbamazepine significantly decreases in intensity and is shifted towards higher frequencies, due to the dissociation of the intermolecular H bonds of CBZ and its inclusion in the CD molecule. The FTIR spectrum of the inclusion complex presents a low intense peak at 1684 cm^−1^ due to the carbonyl group and a broad and low intense peak at 3396 cm^−1^ due to the N–H bond.

**Figure 2:**
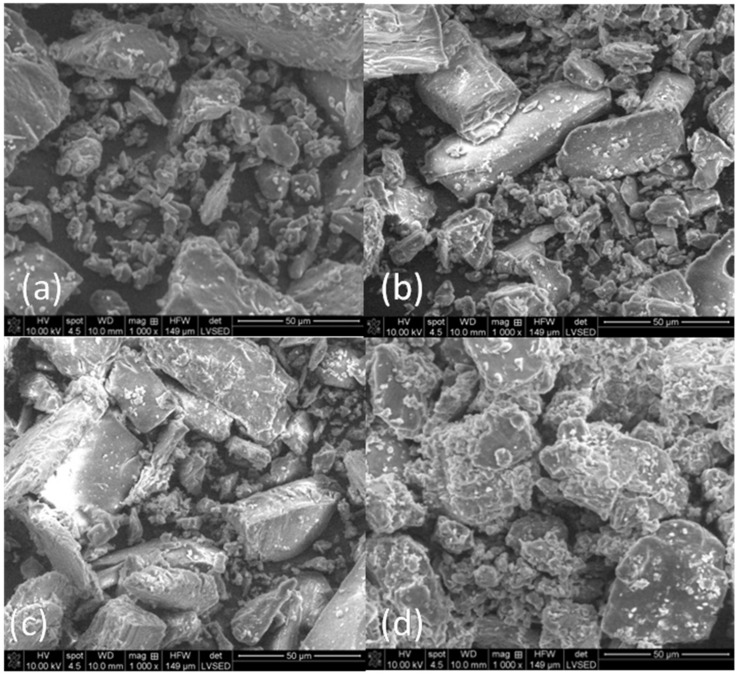
SEM images (**a**) CBZ, (**b**) β-CD, (**c**) CBZ-β-CD physical mixture and (**d**) CBZ-β-CD inclusion complex, at 1000× magnification.

*SEM analysis*. The SEM images of the CBZ, β-CD, physical mixture and CBZ-β-CD inclusion complex are presented in Figure 2. CBZ (Figure 2a) is characterized by the presence of crystalline particles, described as agglomerates of irregular prismatic crystals with various sizes of between 2 and 30 μm. Such crystal shapes of Form III were previously reported in the literature [41].

The SEM image of β-CD shows different shapes of homogenous crystalline particles in polyhedral form [42] with sizes between 5 and 30 μm (Figure 2b).

The SEM image of the CBZ-β-CD physical mixture (Figure 2c) displays the morphologic characteristics of both CBZ and β-CD crystalline particles, with an obvious affinity between the two. The active ingredient crystals can be seen adhering to the surface of CD’s particles. These confirm the onset of the inclusion process that takes place even in their physical mixture.

The SEM picture for the CBZ-β-CD inclusion complex indicates the formation of a new morphological pattern: the mostly spherical shaped particles evidenced in Figure 2d for the inclusion complex are absent in the micrographs of both CBZ (Figure 2a), β-CD (Figure 2b) and their physical mixture (Figure 2c). There is also an obvious decrease in particle size. This reveals a strong interaction between the drug and CD in these systems, indicating that complexation has taken place.

**Figure 3:**
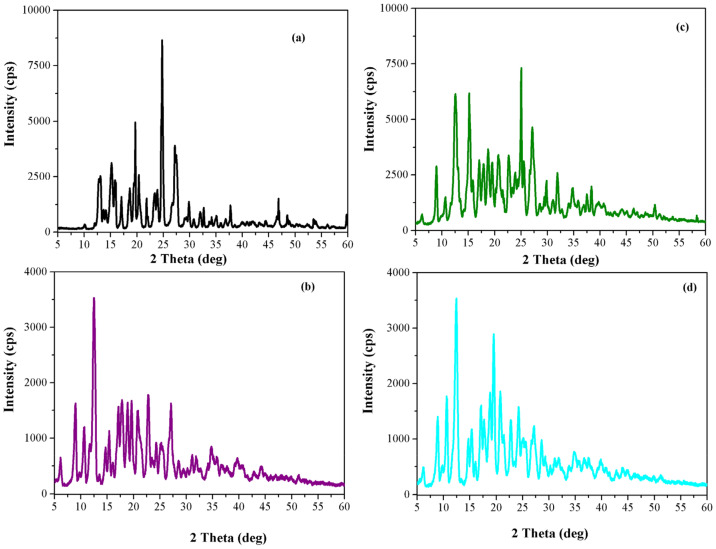
XRD patterns of (**a**) CBZ, (**b**) β-CD, (**c**) CBZ-β-CD physical mixture and (**d**) CBZ-β CD inclusion complex.

*XRD analysis*. Powder X-ray diffraction patterns of the CBZ, β-CD, CBZ-β-CD physical mixture and CBZ-β-CD inclusion complex are presented in Figure 3. X-ray diffractograms of CBZ (Figure 3a) and β-CD (Figure 3b) present many well-defined sharp diffraction peaks, which clearly demonstrate their crystalline nature.

The 2θ diffraction values of the high intensity diffraction peaks of CBZ are 2θ = 13.12°, 15.18°, 15.9°, 19.68°, 24.78°, 27.20° and 31.98°. These characteristic peaks confirm the results of FTIR analysis: the utilized carbamazepine is in polymorphic Form III, in agreement with diffractograms previously reported for this crystalline form [43,44,45]. The X-ray diffraction pattern of β-CD (Figure 3b) also exhibited well-defined peaks with the characteristic peaks observed at 8.98°, 12.50°, 18.90°, 19.60°, 22.78°, 24.33°, 25.48°, 27.1° and 35.7° 2θ diffraction values. According to JCPDS card 00-054-1476, the diffraction lines could be indexed to β-cyclodextrindecahydrate C_42_H_70_O_35_–C_7_H_7_NO_2_·10H_2_O with a monoclinic structure. The XRD pattern of CBZ-β-CD physical mixture (Figure 3c) displays a reduction in the CBZ peaks’ intensities, which indicates a slight decrease in crystallinity. This confirms an interaction between carbamazepine and β-CD (partial inclusion) even in the physical mixture. The XRD diffraction pattern of the inclusion complex (Figure 3d) displayed well-defined peaks at 8.84°, 12.44°, 17.04°, 18.84°, 20.74°, 27.14° and 31.06° 2θ diffraction values. The characteristic pattern that corresponds to the crystalline β-CD is still present with reduced peak intensities, indicating a decrease in crystallinity. The disappearance of the XRD pattern of carbamazepine supports the assumption of a stronger interaction between CBZ and β–CD due to the formation of the inclusion complex.

**Figure 4:**
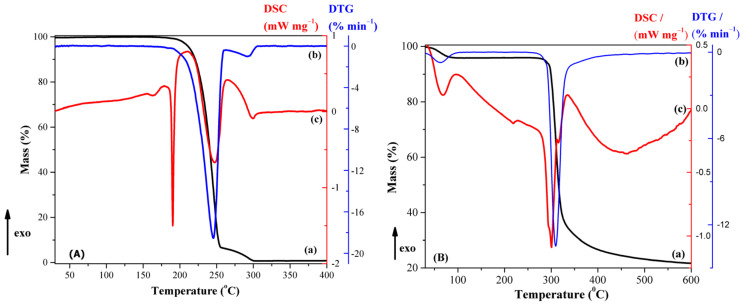
Thermal curves of (**A**) CBZ and (**B**) β-CD, with (a) TG, (b) DTG and (c) DSC (dynamic argon atmosphere, β = 5 °C min^−1^).

*Thermal analysis.* The thermal curves (TG-DTG and DSC) of CBZ are shown in Figure 4A. DSC curves of CBZ (Figure 4A-c) showed an endothermic event at 165.4 °C corresponding to the melting of the polymorphic Form III, the most stable one at ambient temperature, with a heat of fusion of 8.64 J/g. The DSC curve displayed another endotherm at 190.4 °C (92.41 J/g) due to an immediate recrystallization and subsequent melting corresponding to the polymorph Form I. Similar data are reported in the literature [44,46,47].

The thermal curves (TG-DTG and DSC) of β-CD are shown in Figure 4B. The first process, in the 30–105 °C temperature range with a mass loss of 4.18%, is due to the release of weakly bound water molecules from outside and/or inside of β-CD cavity (*T*_DSC_ = 66.5 °C). There is no mass loss in the subsequent, significantly wide 105–265 °C (Δ*T* = 160 °C) temperature range. A small endothermic peak without mass loss appears on the DSC curve of β-CD (*T*_DSC_ = 217 °C), which, according to literature data, is attributed to a reversible structural solid–solid phase transformation [48]. Then, the sample undergoes a rapid and overlapping melting and decomposition processes (mass loss of 75.3% form TG curve, *T_min_* = 300.9 °C and *T_max_* = 335.6 °C from DSC curve). The residue at 600 °C is 20.5%.

**Figure 5:**
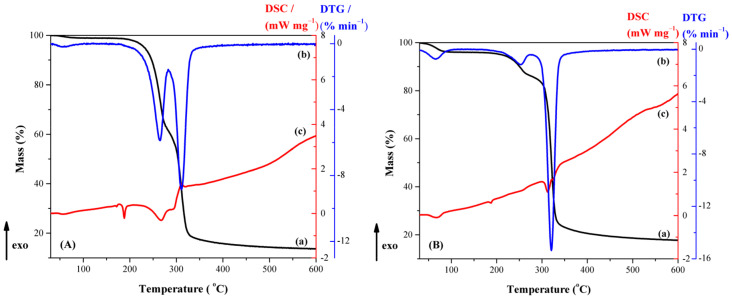
Thermal curves of (**A**) CBZ-β-CD physical mixture and (**B**) CBZ-β-CD inclusion complex, with (a) TG, (b) DTG and (c) DSC (dynamic argon atmosphere, β = 5 °C min^−1^).

**Figure 6:**
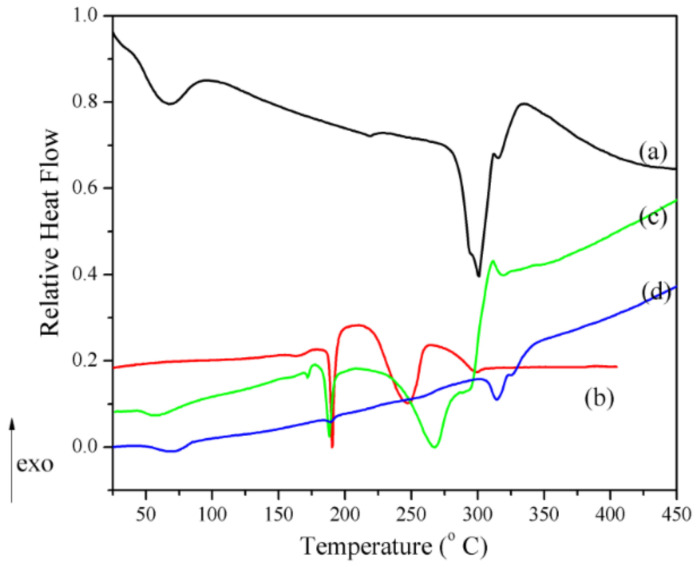
The relative heat flow DSC curves of (a) β-CD, (b) CBZ, (c) CBZ-β-CD physical mixture and (d) CBZ-β-CD inclusion complex.

Thermal curves (TG, DTG and DSC) of the physical mixture and inclusion complex are shown in Figure 5. Several features can be mentioned: (a) the characteristic melting peak of CBZ is shifted from 165.4 °C to 188.23 °C with a significant reduction in its intensity; (b) the peak that corresponds to the β-CD structural solid–solid phase transformation disappeared; (c) the mass loss between 30 and 90 °C, which is attributed to loosely bound water molecules; (d) the degradation of the new phase takes place in two steps with different weights for the physical mixing (*T*_1,DTG_ = 264.1 °C, Δm_1_ = 12.7% and *T*_2,DTG_ = 311.7 °C, Δm_2_ = 62.0%) and kneading procedure (*T*_1,DTG_ = 254.1 °C, Δm_1_ = 37.3% and *T*_2,DTG_ = 320.7 °C, Δm_2_ = 43.5%). To improve the comparison of DSC signals, the “relative heat flow”, RHF, was calculated as described in equation (2) and represented in Figure 6:

(2) $$RHF=\frac{HF\left(T\right)-{HF}_{\min}}{{HF}_{\max}-{HF}_{\min}}$$

where *RHF* is the relative heat flow, *T* is the temperature of the thermal process, *HF*(*T*) is the observed heat flow (the DSC output), *HF*_min_ is the minimum heat flow signal and *HF*_max_ is the maximum heat flow signal [49].

The following features of Figure 6 may be noticed: (i) The presence of a small endothermic peak, associated with the CBZ melting effect, in the thermogram of the physical mixture, and an even smaller one in the thermogram of the inclusion complex, at *T*_DSC_ = 188.23 °C and 188.5 °C, respectively. On the one hand, this clearly signifies a consistent amount of CBZ inclusion into the β-CD cavity, even in the physical mixing procedure; on the other hand, this peak corresponds to some small amount of non-included CBZ for the inclusion complex sample. (ii) The absence of the β-CD phase transition endotherm for the physical mixture and the inclusion complex. (iii) The significant decrease in intensity of the endo-exo thermogram feature associated with the melting-decomposition of β-cyclodextrin in the following order: β-CD > physical mixture >> inclusion complex.

The above-mentioned features confirm the assumption of a loss in the crystalline structure of CBZ and β-CD, due to both physical mixing and kneading method processing, respectively. This process is supported by data obtained in FTIR, XRD and SEM analyses. The partial formation of the inclusion complex even in the physical mixture is also confirmed by the residue, at 600 °C, of both the physical mixture and the inclusion complex, of 13.69% and 17.88%, respectively.

### 3.3. Quantitative Analysis of CBZ

The HPLC method was found to be linear in the range of 3.125–100 μg/mL.

The assessment of the precision of the method resulted in values of the RSD between 0.08 and 0.94%. The bias (%) between the experimental and nominal values of the analytic concentration in the control samples was <2% for all cases. These results demonstrated the accuracy and precision of the chromatographic method.

**Figure 7:**
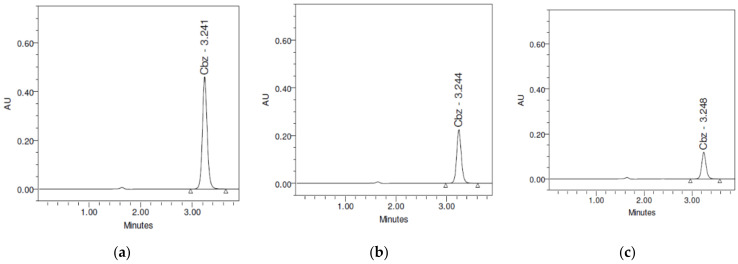
Representative chromatograms of (**a**) the carbamazepine assay; (**b**) a sample obtained in the study of carbamazepine in vitro release kinetics from the CBZ-β-CD chewable tablets in simulated saliva (t = 15 min); (**c**) a sample obtained in the study of carbamazepine in vitro release kinetics from the CBZ-β-CD chewable tablets in 1% LSS (t = 15 min).

The above method was used both for evaluation of carbamazepine content in the chewable tablets containing the CBZ-β-CD inclusion complex, and in the in vitro kinetic studies. Representative chromatograms for these determinations are shown in Figure 7.

### 3.4. In Vitro Release Kinetics

In the first short phase, the thickness of the F-MELT tablets immediately after wetting increased continuously for 5–20 s. After the swelling period, the tablets began to disintegrate rapidly into small particles.

**Figure 8:**
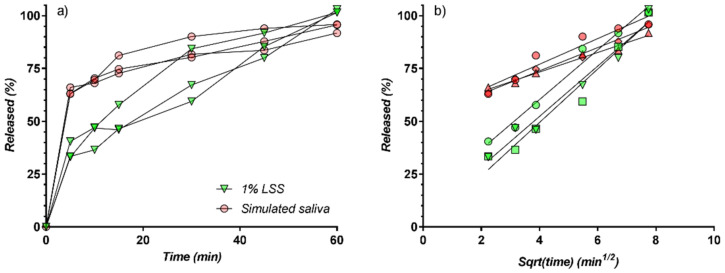
(**a**) Release curves from tablets; (**b**) release curves as function of square root of time.

In Table 1 and Figure 8 all release data and curves, both in SS and LSS, are presented. There are some common characteristics of all curves. There are two distinct phases: the “burst release” in the first 5 min, followed by a smooth portion from 5 to 60 min.

In all cases, release was almost complete at 60 min. In the 5–60 min interval, release curves are well described by a square root time model, suggesting a controlled diffusion mechanism (Figure 8b). Two bundles of parallel straight lines were obtained, corresponding to the SS and LSS media, similar to the release kinetic reported in the case of nimesulide [50] from tablets.

The relatively low variability between curves within the LSS and SS groups justifies further evaluation of only mean curves, corresponding, respectively, to SS and LSS.

**Figure 9:**
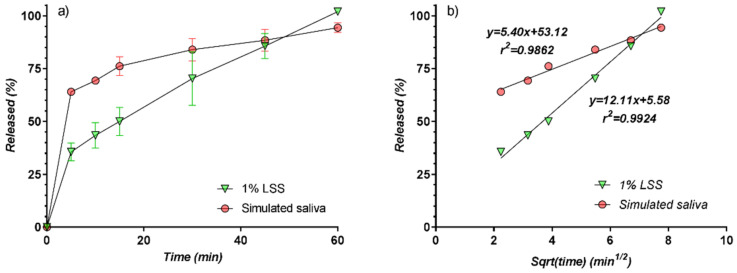
(**a**) In vitro release profiles of CBZ from the chewable tablets containing the CBZ-β-CD complex; (**b**) release curves as function of square root of time.

Comparison of the carbamazepine release profiles in the two-solvent media (LSS and SS) evidenced that dissolution in simulated saliva is much faster than in LSS, with more than 60% of the available substance being released within the first 5 min, and more than 75% within 15 min, compared with only about 50% in 15 min for the LSS profiles. In spite of a very high concentration of LSS, the release profile is superior in the solvent simulating saliva, and the similarity factor *f*_2_ between the two profiles is 34.85, underlying the non-similarity between the carbamazepine release profiles in the two tested media (Figure 9).

**Figure 10:**
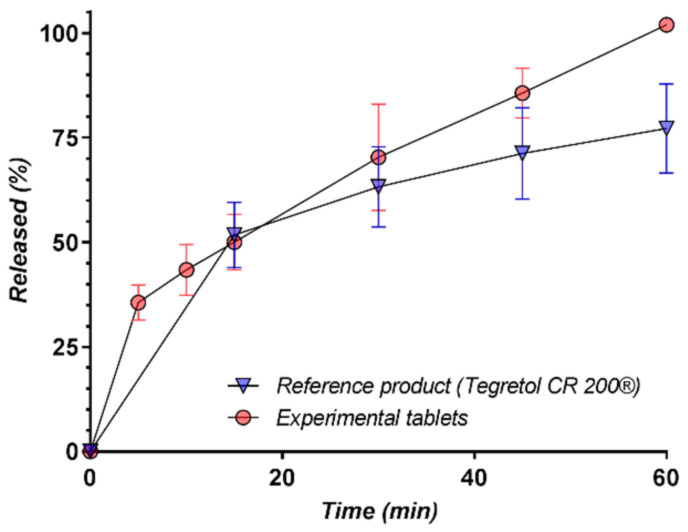
Comparison of the in vitro release profiles of CBZ from the experimental chewable tablets containing the CBZ-β-CD complex and from the commercial reference carbamazepine tablet product in LSS 1%.

In order to estimate the effect of cyclodextrin inclusion on carbamazepine bioavailability, the release of carbamazepine from commercial carbamazepine tablets and from the formulation based on the CD-CBZ complex and the F-Melt excipient was compared (Figure 10).

As can be seen in Figure 10, there is a significant difference (100% vs. 72%) between the released amount at 60 min, which is most probably the consequence of the higher solubility of CBZ-CD in comparison with CBZ (it is worth mentioning that the analytical method measures the entire amount of both CBZ and CBZ-CD).

### 3.5. Modelling of Release Kinetics

As shown above, a representation of the amount of CBZ released by the square root of time led to a linear regression with a sufficiently good correlation coefficient. This is an argument for diffusion-controlled release but, on the other hand, the exclusion of CBZ from the CBZ-CD complex is a concomitant process, a type of “relaxation”, a less diffusive process. This combined mechanism could be described, for example, by a simple heuristically written expression by adding diffusion-controlled and relaxation-controlled drug administration (Equation (3)) [51].

(3) $$M(t)=k_{1}t+k_{2}\sqrt{t}$$

Because, in practice, it is difficult to separate these processes, such behavior has been further described by a widespread expression that has been introduced in the pharmaceutical literature and is known as the “Peppas equation” or “power law” [52]. The power release law has been widely used to describe the first 60% of the release curves [53,54,55].

**Figure 11:**
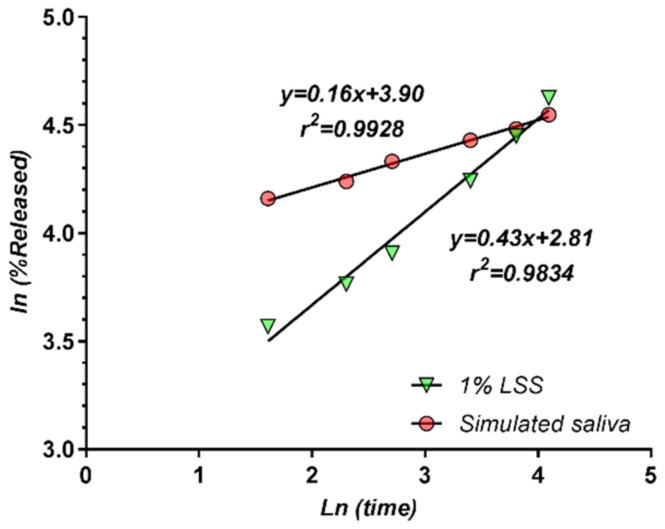
Peppas release model. Fitting of data from 5 min to 60 min.

In our experiment, the release was well described by both models for more than 60%, which is unusual, but not a singular case (Figure 11). In the case of different matrix tablets, it has been found [56] that the power law describes the entire drug release profile.

To select the best model, there are statistical criteria, which consider both the minimum sum of error squares and the number of parameters of the model, and phenomenological criteria [57,58].

A simple comparison between the square root model and the power law model can be considered starting from the estimated value of “n”. In the particular case in which n = 0.5, the power law becomes identical with the square root law. It should be noted that for the release in LSS, a value of n = 0.43 was obtained, which is close to 0.5, thus supporting the square root model and corresponding to a predominant diffusion mechanism. In the case of SS, n = 0.16, which suggests a significant contribution of another mechanism, in parallel with diffusion.

The second phase, well described by both the square root and power law, is a transport controlled mainly by diffusion. In fact, it is possible that this mechanism also characterizes the burst phase. The difference between the two phases could be given by the significant increase in the interface between the formulation matrix and the dissolution medium in the post-disintegration phase.

The ability of a CD to form an inclusion complex is a function of steric factors as well as thermodynamic factors. The driving force for complexation involves, after removal of the water molecule from the hydrophobic cavity and the formation of Van der Waal bonds, hydrophobic interactions, and hydrogen bonds.

A burst release has not been completely ignored so far, but since there is no theoretical justification for this phenomenon, its existence has usually not been “observed”, although it is obvious after a careful examination of the release curves [59].

Especially in recent years, release kinetics containing two phases, with a burst release followed by a slower, diffusion-controlled release—similar to our results—were reported in a series of experimental reports. Curcuminoids’ release from solid lipid nanoparticles, includes a burst release of the curcuminoids followed by a controlled release pattern following the Peppas power model [60]. The same type of release was observed in the case of curcumin embedded in nanocellulose-reinforced chitosan hydrogel [61]. The two-stage release profiles of curcumin from aminosilane-functionalized electrospun poly (N-vinyl-2-pyrrolidone) fibers were fitted well to the Peppas model, indicating a non-Fickian diffusion mechanism for the initial burst release and a Fickian diffusion-controlled mechanism for the sustained release [62]. An initial burst release phase followed by a gradual release phase and good correlation coefficients for the Higuchi model were observed in the case of release of anastrozole from PLGA microparticles in 0.1 N HCl (pH = 1.2) and phosphate buffer (pH = 7.4) [63].

It seems that biodegradable nanoparticles of polycaprolactone represent a matrix that favors a two-phase release kinetic. An initial burst release of ellagic acid followed by Higuchi’s square root pattern in the case of both PLGA and PCL nanoparticles was reported [64]. Burst release is sometimes useful, allowing rapid installation of the effect, but this also involves a risk of increased adverse effects; it is, therefore, sometimes is necessary to reduce the burst phase [65]. Regarding the explanation of the appearance of the burst phase in our experiment, it must be examined in connection with the CBZ-CD complex, as well as the properties of the disintegrating excipient. F-MELT^®^ consists of porous particles with free flow, which are directly compressible. F-MELT^®^-Type C is for faster disintegration needs and has, as its main functional components, microcrystalline cellulose (MCC) and super disintegrating crospovidone. Its main disintegration mechanism is water wicking through the capillary network with only a little swelling. MCC swells notably prior to disintegration [66].

Raman examinations and electron microscopy found that nimesulide is constrained by crospovidone in three main arrangements: an amorphous phase dispersed in the crosslinked molecular network of the polymer, polymer-wrapped nanocrystals, and drug layers composed of micro- and nanocrystals [67].

We do not know whether the same mechanism is applicable to CBZ-CD, but different types of conditions may occur in relation to the povidone cross-matrix, involving different dissociations. The inclusion of CBZ in CD is an association–dissociation balance. The last argument for the reliability of a burst launch was found by DSC and IR analysis, which suggested the existence of a CBZ-free fraction in the structure of the CBZ-CD complex.

As all individual curves suggest, after the immediate, massive release of a free fraction, available at the surface, there is a diffusion-controlled release of molecules into the fragments of the matrix of the tablet. Assuming approximately the same diffusion coefficient *D* for the two types of molecules, one must think of a common diffusion equation, but with different initial and limit conditions.

As for the square root law, it can be obtained in two different sets of initial and boundary conditions. One case is when the front of the solvent enters the matrix step by step and the concentration gradient is in the matrix, as Higuchi considered in 1963:

(4) $$Q(t)=\sqrt{\left(2M-C_{s}\right)C_{s}Dt}$$

where *M* is the total initial quantity, *Q* is the cumulated released quantity and *C_s_* is the saturation concentration of the active substance in the release medium.

Another case, considered in analogy with heat transfer from a thermostat [68,69], is a release given by the formula:

(5) $$Q(t)=c_{s}\frac{2A}{\sqrt{\pi }}\sqrt{Dt}$$

where the released quantity *Q* is proportional with the surface *A*, concentration (constant at the surface) *c_s_*, and with the square root of the time.

The kinetics of CBZ release from a physical mixture of β-CD and from two CBZ-CD complexes from hydroxypropylmethyl cellulose matrix (HPMC) slow-release tablets was modeled using different mathematical equations [70]. The data were fitted by four mathematical models: zero-order kinetics, first-order, Higuchi and Weibull. The Higuchi model was able to match the release from the complexes, but not from the physical mixture. The Weibull model parameters were more sensitive to the differences between the two sets of kinetic release data. HPMC and commercial Tegretol CR 200^®^ tablets were tested in 1% sodium lauryl sulfate [14]. In the case of HPMC matrices, the tablets underwent gelation and erosion, and very slow dissolution of the matrix followed a zero-order kinetics in the range of 0–300 min. CBZ release from Tegretol CR 200^®^ was faster compared to matrices and exhibited distinct kinetic behavior. It should be noted that in those experiments, burst release occurred only in the case of Tegretol tablets.

One final issue that needs to be discussed is that the release was higher in SS than in LSS. As observed in other studies [4], the effect of surfactants on the dissolution kinetics of poorly soluble drugs is higher in terms of release extent and lower in terms of release rate. In the case of soluble drugs, the effect is low on both the rate and the extent [71].

## 4. Conclusions

The properties of a novel inclusion complex of CBZ and β-CD were investigated as a promising candidate for the preparation of orodispersible tablets. The studied inclusion complex was obtained in a molar ratio of 1:1, using the kneading method of complexation, in a solid state. For comparison, a physical mixture between CBZ and β-CD, in the same molar ratio, was prepared and analyzed. The SEM images of the complex show aggregation into irregularly shaped more amorphous particles in which the morphology of both components has disappeared. This remarkable change indicates the formation of a binary inclusion complex between CBZ and β-CD. This is confirmed by FTIR, XRD and thermal analysis investigations. The formation of the CBZ-β-CD inclusion complex results in important modifications of the pharmacological properties (solubility, absorbability, and consequently bioavailability) of the active ingredient. These modifications may have a significant impact on the biological effects of the drug. Therefore, the natural development of this study was the preparation and characterization of several formulations of orodispersible tablets containing this new CBZ-β-CD inclusion complex. Tablet formulations that included the CBZ-CD complex and the super-disintegrant F-Melt^®^ release CBZ faster compared to commercial CBZ Tegretol tablets. The kinetic release of total carbamazepine from tablets containing CBZ-CD and super-disintegrant F-Melt, in both SS and LSS, follows two stages: (i) a burst release in the initial minutes and (ii) a slower release at different time intervals up to 60 min. The release in the second phase is well described by the Higuchi and Peppas models, which support a diffusion-controlled release combined with some phenomena of swelling and relaxation of the matrix generated by the crospovidone component of the F-Melt excipient.

## Figures and Tables

**Table 1:** The individual and mean values of release in LSS and SS.

Time (min)	LSS 1%			Simulated Saliva		
	Mean	SD	RSD (%)	Mean	SD	RSD (%)
5	35.60	4.19	11.78	64.04	1.72	2.69
10	43.42	6.02	13.86	69.34	1.15	1.65
15	50.01	6.63	13.26	76.15	4.43	5.82
30	70.29	12.73	18.12	83.99	5.31	6.32
45	85.67	5.90	6.89	88.44	5.19	5.86
60	101.99	0.84	0.82	94.42	2.29	2.42
